# Second-Line HIV Therapy Outcomes and Determinants of Mortality at the Largest HIV Referral Center in Southern Vietnam

**DOI:** 10.1097/MD.0000000000001715

**Published:** 2015-10-30

**Authors:** Vu Phuong Thao, Vo Minh Quang, Marcel Wolbers, Nguyen Duc Anh, Cecilia Shikuma, Jeremy Farrar, Sarah Dunstan, Nguyen Van Vinh Chau, Jeremy Day, Guy Thwaites, Thuy Le

**Affiliations:** From the Wellcome Trust Major Overseas Programme, Oxford University Clinical Research Unit (VPT, MW, NDA, JF, JD, GT, TLE); Hospital for Tropical Diseases, Ho Chi Minh City, Vietnam (VMQ, NVVC); Hawaii Centre for AIDS, University of Hawaii at Manoa, Honolulu, Hawaii (CS, TLE); and Peter Doherty Institute for Infection and Immunity, The University of Melbourne, Melbourne, Australia (SD).

## Abstract

The growing numbers of HIV-infected patients requiring second-line antiretroviral therapy (ART) in Vietnam make essential the evaluation of treatment efficacy to guide treatment strategies.

We evaluated all patients aged ≥15 years who initiated second-line ART after documented failure of first-line therapy at the Hospital for Tropical Diseases in Ho Chi Minh City. The primary outcome was time from second-line ART initiation to death, or to a new or reoccurrence of a WHO-defined immunological or clinical failure event, whichever occurred first. Risks of treatment failure and death were evaluated using Cox proportional hazards modeling.

Data from 326 of 373 patients initiating second-line ART between November 2006 and August 2011 were included in this analysis. The median age was 32 years (IQR: 28–36). Eighty one percent were men. The median CD4 count was 44 cells/μL (IQR: 16–84). During a median follow-up of 29 months (IQR: 15–44), 60 (18.4%) patients experienced treatment failure, including 12 immunological failures, 4 WHO stage IV AIDS events, and 44 deaths (13.5%). Sixty percent of deaths occurred during the first 6–12 months. The Kaplan–Meier estimates of treatment failure after 1, 2, 3, and 4 years were 13.1% (95% CI: 9.2–16.8), 18.6% (95% CI: 14.0–23.1), 20.4% (95% CI: 15.4–25.1), and 22.8% (95% CI: 17.2–28.1), respectively. Older age, history of injection drug use, lower CD4 count, medication adherence <95%, and previous protease inhibitor use independently predicted treatment failure.

While treatment efficacy was similar to that reported from other resource-limited settings, mortality was higher. Early deaths may be averted by prioritizing second-line therapy for those with lower CD4 counts and by improving treatment adherence support.

## INTRODUCTION

The availability of low-cost fixed-dose combination antiretroviral drugs has enabled rapid scale-up of antiretroviral therapy (ART), resulting in substantial reduction in morbidity and mortality due to HIV in resource limited countries.^1–3^ The World Health Organization (WHO) estimates that 16.8 million adults and children in low and middle-income countries will be on ART in 2016; among them 5% will be on second-line therapy.^4^ This represents a more than 50% increase in ART coverage over the past 5 years. Despite generic production for resource-limited countries, a second-line regimen containing ritonavir-boosted lopinavir (LPVr) costs 6 times that of a first-line regimen.^5^ In most low and middle-income countries, second-line therapy is the last option for patients failing treatment with drug resistance. As third-line therapy is forbiddingly expensive and is unavailable in resource-limited countries, it is imperative for national programmes in these settings to maximize the efficacy and durability of second-line therapy.

Vietnam is among the countries with the highest HIV burden in Asia with an estimate of 280,000 people living with HIV.^6^ Nearly 90,000 people were on ART as of 2014, and an estimated 3% were on second-line therapy.^6^ The HIV system in Vietnam is undergoing a critical transition from an international-donor to a national-funding approach that integrates with the national health insurance programme.^7^ Outcome data on second-line therapy in Vietnam are lacking, but are important for the national programme to devise treatment strategies and to forecast treatment options beyond second-line therapy. In this study, we investigate second-line therapy outcomes and factors that determine therapy failure and death at the largest HIV referral centre in southern Vietnam.

## METHODS

### Study Design and Setting

This was a retrospective analysis of adult patients who switched to second-line therapy in a cohort of over 4000 patients on the national ART programme at the Hospital for Tropical Diseases (HTD) in Ho Chi Minh City (HCMC). This is the largest primary and referral center for HIV care in southern Vietnam (population around 45 million). The national ART programme began providing free antiretroviral drugs through international funding support in 2003. First-line therapy consisted of zidovudine (AZT) or stavudine (d4T) in combination with lamivudine (3TC) and nevirapine (NVP). Prior to the availability of efavirenz in 2004, cases of NVP-related toxicity were switched to indinavir (IDV). Second-line therapy became available in 2006 initially including abacavir (ABC), didanosine (ddI), and nelfinavir (NFV). In 2007, LPVr replaced NFV, and in 2009 tenofovir (TDF) and 3TC replaced ABC and ddI as the nucleotide reverse transcriptase inhibitor (NRTI) backbone.^8^

### ART Monitoring

ART was monitored using immunological and clinical failure criteria based on the WHO's guidelines for settings without routine viral load monitoring.^9,10^ Patients were required to come to the clinic monthly for clinical evaluation and medication pick-up. CD4 count was measured every 6 months. HIV viral load was tested at the time patients were diagnosed with immunological or clinical failure and was confirmed with repeat testing. HIV viral load was performed using a generic real-time PCR assay (Biocentric, Bandol, France) with a limit of detection of 250 copies/mL.^11^ Virological failure was defined as confirmed HIV RNA levels ≥5000 copies/mL as per WHO guidelines during the study period.^10^ HIV genotyping was performed to evaluate for drug resistance prior to therapy switch using a published in-house assay^12^ on the Beckman Coulter CEQ 8000 platform. Both HIV viral load and genotyping tests were performed at the Pasteur Institute, a WHO-accredited HIV reference laboratory, in HCMC.

### Study Population

We included all HIV-infected patients aged ≥15 years who initiated second-line therapy due to documented immunological and/or clinical failure of first-line therapy. Patients who were alive and well but had been on second-line therapy for <6 months, and those without documented treatment failure to first-line therapy, were excluded. The study was approved by the Scientific and Ethical Committee of the HTD.

### Outcome Measurements

The primary outcome was treatment failure and was defined as time from second-line ART initiation to death, or to a new or reoccurrence of an immunological or a clinical failure event, whichever occurred first. Immunological failure was defined by the WHO as a decrease of CD4 count to or lower than baseline, a decrease of >50% of peak CD4 value while on treatment, or a persistent CD4 count of <100 cells/μL after at least 6 months of continued ART. Clinical failure was defined as new occurrence or reoccurrence of a WHO stage IV disease.^10^ The secondary outcome was time to death.

### Data Collection

Routinely collected clinical and laboratory data were recorded on a standardized form and included demographic information, history of injection drug use (IDU), antiretroviral drug timeline, WHO stage 4 AIDS events, 6-month serial CD4 counts, HIV viral load, and HIV genotype when these were available, ART adherence evaluation, deaths, and causes of death.

### Antiretroviral Therapy Adherence Evaluation

ART adherence counseling was provided to patients pre- and post-second-line ART initiation according to standard of care. Adherence was routinely assessed by the clinicians according to the MOH guidelines and was recorded either as an estimated percentage of pills taken, or as a qualitative assessment of “good,” “average,” or “poor,” corresponding to ≥95%, 80% to 94%, or <80% adherence, respectively.^13^ Additionally in patients who were in active follow-up, adherence was prospectively evaluated using a simple self-reported Visual Analogue Scale (VAS).^14^ This VAS has been shown to be as reliable as other methods such as pill-count and 3-day recall self-report, yet much simpler to administer.^15^

For analysis, suboptimal adherence was defined as having at least 1 adherence score of <95% by pill count, by the VAS, and/or receiving at least 1 qualitative adherence assessment of “average” or “poor” over a 6-month period preceding an outcome event or preceding the time of study assessment in patients who had not had an event.

### Statistical Analysis

The cumulative incidence of treatment failure and failure rates after 1, 2, 3, and 4 years and corresponding 95% confidence intervals were calculated using the Kaplan–Meier method. The Cox proportional hazards model was used to analyze the time to treatment failure (composite primary endpoint) and the time to death (secondary endpoint). Patients who were transferred to other provincial or district clinics while on second-line therapy had been judged by doctors to be clinically and immunologically stable before the transfer. For the analysis, event-free transferred patients were censored at the time of transfer (primary analysis). Alternatively, assuming the transferred patients were doing well clinically and immunologically on therapy, we treated them as censored at the time-point where their last monthly follow-up visit would have been had they not been transferred (sensitivity analysis to assess potential informative censoring).

The following predefined covariates were included in the model: age at second-line therapy initiation, history of IDU (yes/no), CD4 cell count, and (log10-transformed) HIV RNA viral load at second-line therapy initiation, second-line therapy delay (defined as time from first detection of immunological or clinical failure to time of second-line therapy initiation), history of protease inhibitor (PI) use, and an overall measure of therapy adherence (<95% vs. ≥95%). The chosen covariates have been shown to be associated with poor ART outcome.^16–21^ Although recommended by the WHO guidelines,^10^ not all patients in the cohort had a viral load performed to document virological failure to first-line therapy prior to second-line therapy initiation. We performed a sensitivity analysis including only patients with documented virological failure to first-line therapy (confirmed HIV RNA levels ≥5000 copies/mL). The proportional hazards assumption was assessed by examining plots of weighted Schoenfeld residuals and by formal testing. There was strong evidence of nonproportional hazards for the effect of CD4 cell count at second-line therapy initiation on treatment failure (*P* = 0.001 univariate analysis, *P* = 0.0002 multivariable analysis). To account for this, we decided to model a time-varying effect on the hazard of treatment failure with separate effects for the first year of follow-up and subsequently. There was no clear evidence for nonproportional hazards between any other covariates and the primary or secondary endpoint (all univariate *P* > 0.13). Both univariate and multivariable Cox regressions were performed.

Data were analyzed based on multiple imputations of missing data and on a complete-case analysis. To avoid bias, the imputation algorithm included the endpoints [event time *T* and Nelson-Aalen estimator H(T)].^22^ All reported confidence intervals are 2-sided 95% intervals and analyses were performed with the statistical software R version 2.15.0,^23^ and the companion R package mice version 1.2.5 (for multiple imputation).^24^

## RESULTS

### Study Population and Baseline Characteristics

A total of 373 patients aged ≥15 years initiated second-line therapy between November 2006 and August 2011. Forty-seven patients were excluded from the analysis, including 43 who had received second-line therapy for <6 months and 4 who had switched to second-line therapy because of treatment intolerance. The remaining 326 patients had documented treatment failure to first-line therapy [with confirmation of virological failure in 305 (94%) patients] and were included in this study. Approximately 50% of patients came from HCMC; the rest from the remaining 17 southern provinces of Vietnam. The median duration of first-line ART treatment was 33 months (IQR: 21–44). The characteristics of the 326 patients at the time of initiation of second-line therapy are summarized in Table 1. The median CD4 count was 44 cells/μL (IQR: 16–84) and the median HIV RNA was 5.1 log copies/mL (IQR: 4.6–5.6). The median time of second-line therapy delay was 9 months (IQR: 5–15).

**TABLE 1:**
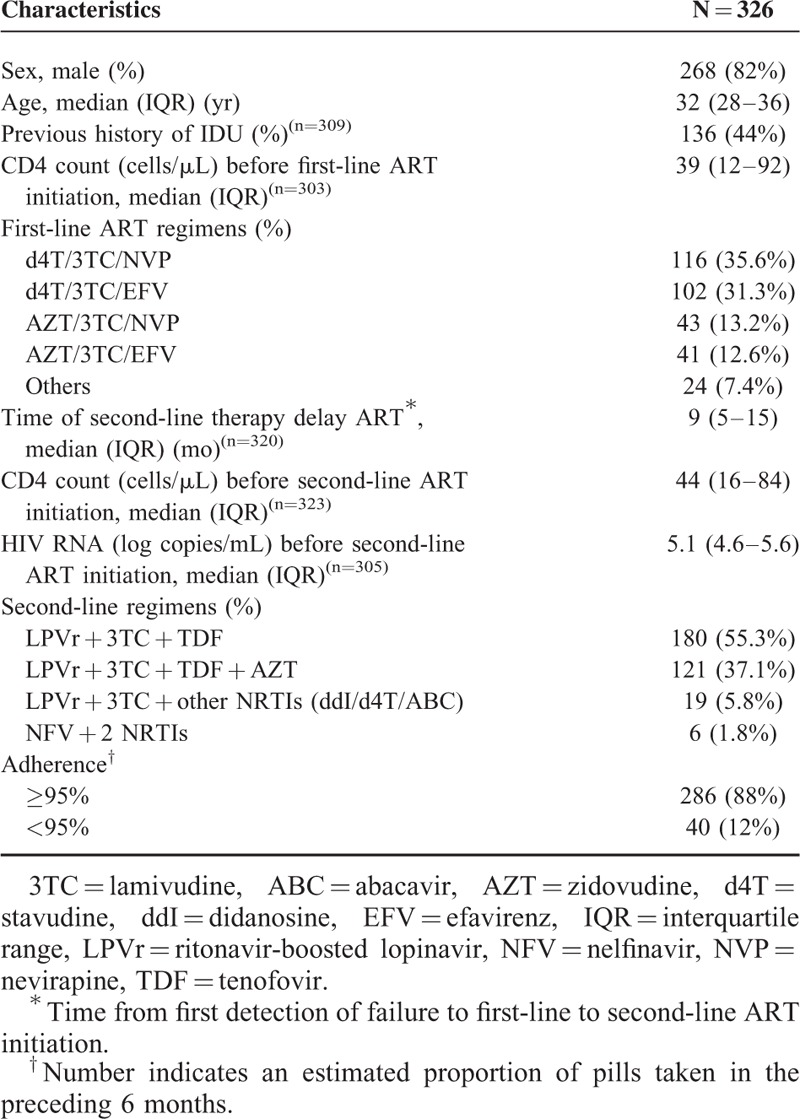
The Characteristics of 326 Patients Starting Second-Line ART at the Hospital for Tropical Diseases in Ho Chi Minh City

### Drug Resistance Patterns in Patients Failing First-Line ART

HIV genotyping was performed for 246 of 326 (75.5%) patients who failed first-line therapy. Mutations conferring high-level resistance to NRTIs were detected in 238 of 246 patients (96.7%), to nonnucleotide reverse transcriptase inhibitors (NNRTIs) in 229 of 246 (93.1%), and to PIs in 6 of 246 (2.4%). Resistance mutations to both NRTIs and NNRTIs were present in 226 of 246 patients (91.9%) and to all 3 drug classes in 5 of 246 patients (2.0%). The most common NRTI mutations were M184I/V (85.4%), thymidine analog mutations (TAMs) M41L, D67N, K70E/R, T215F/Y, and K219E/Q (30–55%), Q151 M (21.1%), and K65R (14.6%). Two patients had a T69 insertion mutation. The most common NNRTI mutations were Y181C/I/V (45.5%), G190A/S (41.9%), and K103N (31.3%). The most common PI mutations were I54 V (2.4%), M46I/L (2.8%), V82A (2.0%), and L90 M (1.2%).

### Predicted Resistance to Second-Line ART Regimen

The predicted susceptibility to the national second-line regimen containing TDF, 3TC, and LPVr was evaluated for the 246 patients who had genotype results using the Stanford HIV Drug Resistance Database (access date: April 9, 2015). Intermediate to high-level resistance to TDF was present in 161 of 246 (65.4%), to 3TC in 230 of 246 (93.5%), and to LPVr in 5 of 246 (2.0%).

### Second-Line ART Outcome

**FIGURE 1:**
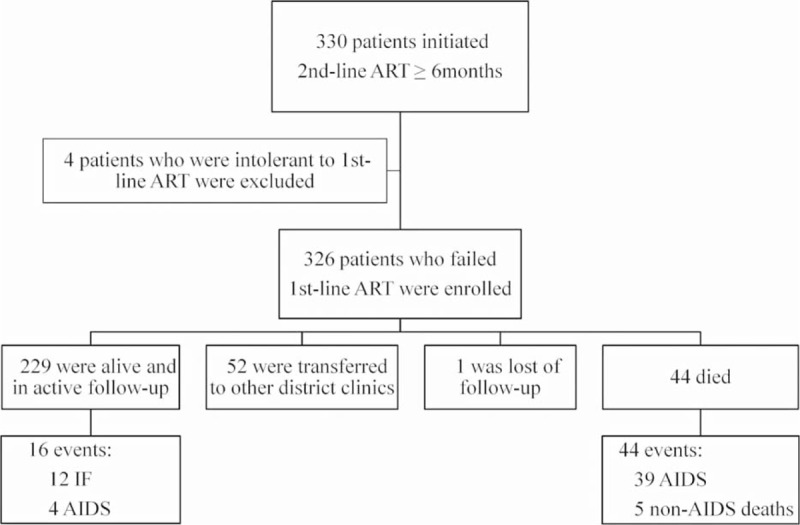
Flowchart of study population and outcome events.

**FIGURE 2:**
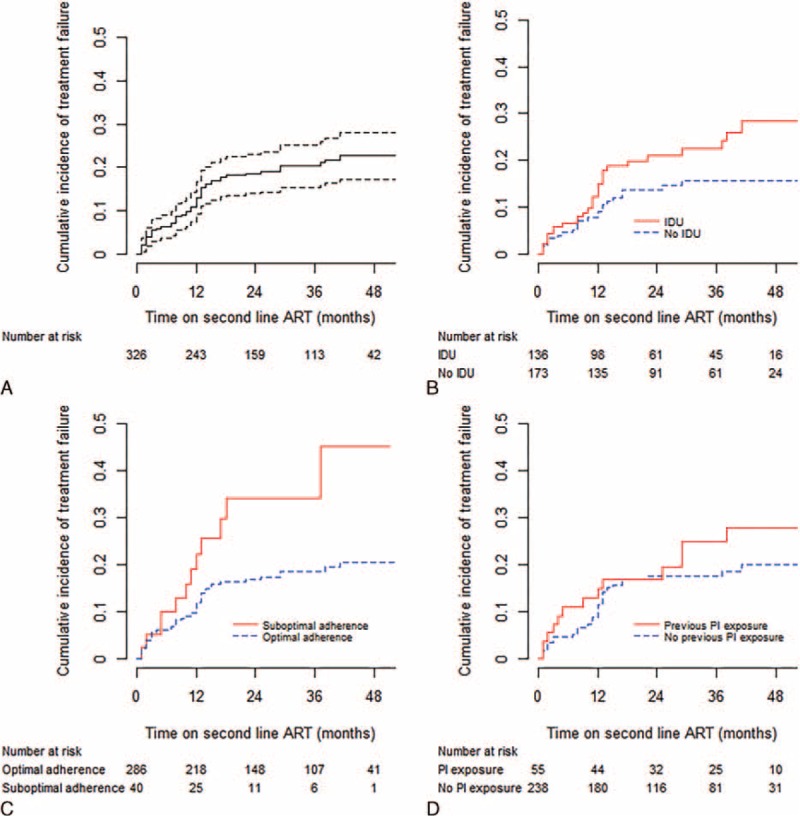
The cummulative incidence of treatment failure on second-line antiretroviral therapy over time. A, Treatment failure in all patients in the cohort. The dotted lines represent the point-wise 95% confidence interval. B, Treatment failure in patients with and without history of injection drug use. C, Treatment failure in patients with suboptimal and optimal antiretroviral adherence. D, Treatment failure in patients with previous and no protease inhibitor exposure.

In total, 320 patients (98.2%) received LPVr in combination with 2 NRTIs and 6 patients received NFV with 2 NRTIs (Table 1). One hundred twenty-one patients (37.1%) also received AZT; this was chosen by clinicians who believed that an AZT-containing regimen might reduce the likelihood of developing the TDF-signature-resistance-mutation K65R, and thereby preserve the potency of the second-line regimen. Suboptimal adherence was observed in 43 patients (13.2%). During a median follow-up of 29 months (IQR: 15–44), 52 patients (16.0%) were transferred to other clinics as part of the government's efforts to decentralize HIV care; 1 was imprisoned and was lost to follow-up, 44 (13.5%) died and the remaining 229 (70.2%) were in active follow-up. Sixty (18.4%) patients experienced treatment failure, including 12 immunological failures, 4 WHO stage IV AIDS events, 39 AIDS-related deaths, and 5 non-AIDS deaths (Fig. 1). The cumulative incidence of treatment failure and corresponding 95% confidence interval are shown in Figure 2A. The Kaplan–Meier estimates of the risk of treatment failure by 1, 2, 3, and 4 years were 13.1% (95% CI: 9.2–16.8), 18.6% (95% CI: 14.0–23.1), 20.4% (95% CI: 15.4–25.1), and 22.8% (95% CI: 17.2–28.1), respectively. The median CD4 counts after 1, 2, 3, and 4 years were 234 cells/μL (IQR: 166–338), 353 cells/μL (IQR: 227–465), 393 cells/μL (IQR: 255–514), 473 cells/μL (IQR: 347–574), respectively.

### Predictors of Second-Line ART Failure

The 7 covariates entered in the Cox model are listed in Table 2. The most frequently missing covariates were history of PI use (10% missing), viral load (6% missing), and IDU history (5% missing); other covariates were missing in ≤2% of patients. The results of the univariate and multivariate Cox regression analyses are shown in Table 2. Lower CD4 count and suboptimal adherence predicted treatment failure in both univariate and multivariate analyses. However, lower CD4 count affected the rate of treatment failure only during the first year of second-line therapy and not thereafter. Older age, history of IDU, and history of PI use did not predict treatment failure in the univariate analysis, but in the multivariate analysis they became statistically significant predictors of treatment failure. Multivariate analysis shown in Table 2 was based on multiple imputations of missing data; however a complete-case analysis gave highly consistent results (data not shown). A sensitivity analysis with informative censoring of the 52 transferred patients was performed (ie, assuming the transferred patients continued to do well clinically and immunologically on therapy, with censoring occurring at the time-point where their last monthly follow-up visit would have been had they not been transferred), and the results were also consistent (data not shown). A second sensitivity analysis excluding 21 (6%) patients without virological confirmation of treatment failure to first-line therapy was performed, and the results were again consistent. Older age, history of IDU, lower CD4 cell count, suboptimal adherence, and history of PI use were independent predictors for treatment failure in the multivariate analysis, ORs (95% CI), *P* values: 2.34 (1.53–3.59), *P* < 0.001; 3.09 (1.50–6.38), *P* = 0.002; 0.54 (0.32–0.89), *P* = 0.016); 3.07 (1.52–6.22), *P* = 0.002; and 2.22 (1.06–4.67), *P* = 0.035), respectively.

**TABLE 2:**
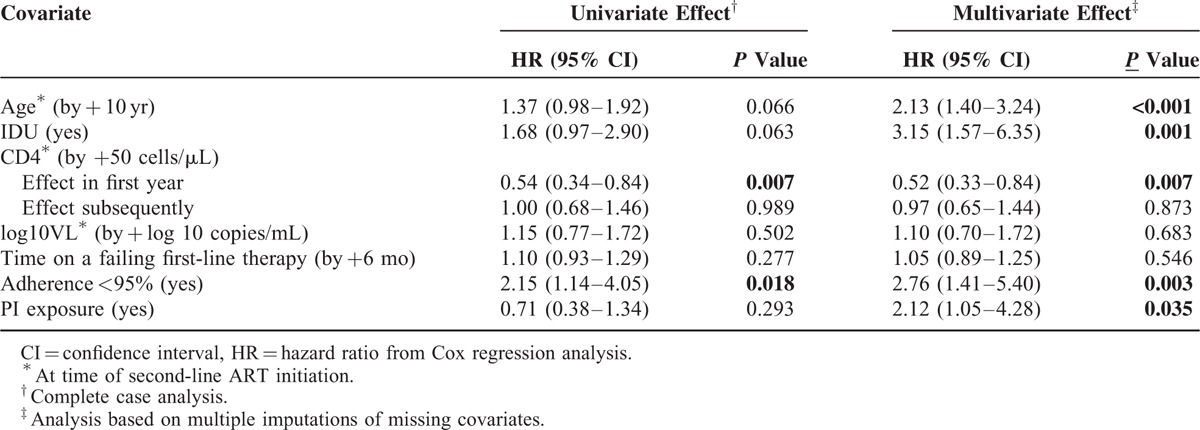
The Impact of Covariates on Treatment Failure

The cumulative incidence of treatment failure among patients according to IDU, treatment adherence, and PI is shown in Figures 2B–D, respectively. We performed an exploratory univariate analysis of the impact of adding AZT as a 4th drug to second-line regimen using Cox proportional hazard modeling, and a statistically significant effect on treatment outcome was not observed (HR: 1.12, 95% CI: 0.50–1.62, *P* = 0.715).

### Causes and Predictors of Death

Deaths occurred in 44 patients (13.5%) and accounted for 73.3% of failure events; 39 were AIDS-related deaths, and 5 were unknown or non-AIDS-related deaths. The median time to death was 9 months (IQR: 3–22), with 26 deaths (59.1%) occurring within the first 6 to 12 months. The causes of AIDS-related deaths included microbiologically confirmed tuberculosis (13, 29.5%), *Pneumocystis jiroveci* pneumonia (4, 9.1%), candida esophagitis (4, 9.1%), cryptococcal meningitis (2), *Penicillium marneffei* infection (2), herpes simplex (2), cytomegalovirus (2), toxoplasmosis (2), hepatitis C-related liver failure (2), renal failure of unclear etiology (2), nontyphoid salmonella sepsis (1), and AIDS-associated wasting (8, 18.2%). Univariate and multivariate Cox regression analyses were performed to determine predictors of death. Consistent with the results of the primary outcome analysis, lower CD4 count (multivariate HR: 1.75, 95% CI: 1.11–2.70, *P* = 0.015) and suboptimal adherence (multivariate HR: 3.41, 95% CI: 1.59–7.30, *P* = 0.002) predicted death in both univariate and multivariate analyses. Age and history of IDU did not predict death in the univariate analysis, but in the multivariate analysis older age and history of IDU became significant predictors of death (HR: 1.95, 95% CI: 1.22–3.11, *P* = 0.005 and HR: 2.30, 95% CI: 1.05–5.05, *P* = 0.037, respectively).

## DISCUSSION

To our knowledge, this is the first study to systematically evaluate the outcomes of second-line ART in patients who fail first-line therapy in Vietnam according to the WHO's immunological and clinical criteria. Despite the profound level of immune deficiency in this patient cohort (median CD4 count: 44 cells/μL), high viral replication (median HIV RNA: 5.1 log copies/mL), and extensive resistance to second-line NRTI backbone (93.5% resistance to 3TC, 65.4% resistance to TDF) at the time of treatment switch, treatment failure rates are similar to studies in comparable settings.^16,18,19,21,25^ A 27-cohort study comprising 632 patients from Africa and Asia reported a failure rate of 28% over 2 years.^16^ Treatment failure in that study was defined as the first diagnosis of clinical, immunological or virological failure, or death. Only 4 of these 27 centers had routine viral load monitoring. These failure rates reflect the reality of HIV care in resource-limited settings. Our treatment failure rates are lower when compared with studies that use virological failure as the measure of outcomes. A meta-analysis of 2035 patients from 19 cohorts across low-income and middle-income countries reported failure rates of 22% to 38% after 6 to 36 months on second-line ART.^25^ Although an outcome measure relying on CD4 count and clinical evaluation can underestimate virological failure rates, the long-term clinical and health economic benefits of routine viral load monitoring still need to be determined in resource-poor settings.

While the overall treatment failure rates were similar to equivalent settings, the mortality in our cohort was higher: 13.5% versus an estimate of 5% in the 27-cohort study from Africa and Asia.^16^ The median time to death was shorter, 9 months (IQR: 3–22) versus 15 months (IQR: 12–26).^16^ Death accounted for 73% of failure events, and 90% of deaths were due to AIDS-related infections. Using multivariate analysis, death during the first year was predicted by lower CD4 counts at second-line ART initiation. The median CD4 count and HIV viral load at second-line ART initiation were 44 cells/μL and 5.1 log copies/mL, respectively. This compares with median CD4 counts >100 cells/μL and HIV viral load ranging from 3.9 to 4.8 log copies/mL in other African and Asian cohorts.^17,19–21,26,27^ The lower CD4 count likely explains the higher mortality observed in our patients and suggests second-line therapy delay plays a role. When comparing to studies from other resource-limited countries, therapy delay in our cohort was longer, median 9 (range: 5–15) versus 5 (range: 1–8) months.^17,18,20,26^

Therapy delay can be explained by programmatic reasons not unique to Vietnam. Without viral load monitoring, it can take months to years for a patient who fails treatment virologically to manifest failure immunologically and clinically. The process of defining treatment failure in Vietnam may be too conservative. Immunological failure is usually confirmed with a repeat CD4 measure in 3 to 6 months. Cases of confirmed immunological and/or clinical failure are referred to a panel of experts located in centers that have access to viral load and drug resistance testing. The referral process, albeit necessary in some circumstances, further delays the onset of therapy switch. When the relationship of treatment delay and CD4 count was assessed with respect to therapy failure and death in our study, only lower CD4 count predicted therapy failure and death. The likely explanation for this is that these 2 variables are interdependent or have a causal relationship, that is, treatment delay directly results in a decline in CD4 count. In our cohort, the CD4 count is likely the stronger variable that drives the outcomes. When these same variables were evaluated by Levison et al. in a study using virological failure as outcome, they found that therapy delay and not CD4 count predicted virological failure, and for every month a patient remained on a failing first-line ART regimen there was a 7% increase in risk of lack of virological suppression.^20^ The longer a nonsuppressive ART regimen is given, the higher the chance of developing accumulation of drug resistance mutations, impairing the efficacy of current as well as future ART options.

In our cohort older age, history of IDU, PI exposure, lower CD4 count, and suboptimal adherence were independent predictors of treatment failure in both complete-case and multiple imputation analyses. With the exception of PI exposure, these variables remained independent predictors of death. These findings are consistent with the published literature on first-line ART failure worldwide.^28–34^ Older age at ART initiation has been linked to poor immunological recovery, loss to follow-up, and death in ART cohorts in Zambia and South Africa.^29,31^ CD4 count and treatment adherence are established risk factors for first-line ART failure and are also found to be independent predictors of second-line ART failure in the analysis of 27 ART programmes across Africa and Asia.^16^ PI exposure has been associated with risks of virological failure in Cambodia and India.^35,36^ Treatment adherence in our study was assessed at multiple time points, suggesting that suboptimal adherence at anytime during therapy predicts treatment failure. This finding is similar to other second-line cohorts from South Africa, Malawi, and Thailand,^17,19,21^ highlighting the importance of ongoing patient education and adherence support in improving treatment outcomes. The history of IDU predicted treatment failure independently from treatment adherence. This effect is likely multifactorial and likely involves cofactors that are not measured in this study including nutritional status, social economic status, and hepatitis B and/or C coinfection.

Coinfection with tuberculosis in patients failing ART is common in countries where tuberculosis is highly endemic. In our study, microbiologically confirmed tuberculosis accounted for the majority of AIDS-related deaths (30%). HIV and tuberculosis coinfection is a common reason for clinicians to delay second-line ART initiation because of the rifampicin and PI drug–drug interactions via CYP450 metabolism pathway. The WHO recommends rifabutin in place of rifampicin for patients who need tuberculosis treatment while on PI therapy. Unfortunately rifabutin is not yet available in Vietnam. A super-boosted dose of ritonavir (400 mg twice daily) is therefore recommended.^37^ The safety data of this super-boosted ritonavir regimen in HIV-associated tuberculosis are very limited,^38,39^ and significant side effects and hepatic toxicity have been reported in healthy volunteers.^40^ Therefore, many clinicians in developing countries defer second-line therapy until the rifampicin-containing phase of tuberculosis treatment is complete. This delay may explain the significantly higher treatment failure rate (51%) in patients with tuberculosis coinfection in our cohort.

Our study has limitations. This is a single-center study; therefore, the criticism is that the patients may not be representative of the entire adult population on second-line therapy in Vietnam. However, the HTD is the largest center for HIV in Vietnam and is the primary provider of second-line therapy for patients in southern Vietnam. Half of the patients in this cohort come from the 17 southern provinces of Vietnam, representing a wide selection of patients. Further this study is the largest second-line therapy cohort in Southeast Asia, allowing for robust analyses of clinical outcomes. Another limitation is that clinical data are not prospectively collected. However, all study variables were consistently assessed in a standardized way in accordance with the national guidelines. We expect the quality of data is approaching that of a prospective study. Lastly, the attrition rate was high (16%); however, this is largely due to transfer of care (52 patients). Only 1 patient was lost to follow-up.

In summary, this is the first study to report the outcomes of second-line ART with a LPVr-based regimen in Vietnam. The overall treatment failure rate using immunological and clinical criteria is 18.4% after a median follow-up of 29 months. Early AIDS-associated death is the main result of treatment failure and is predicted by older age, history of IDU, lower CD4 count at therapy switch, and medication adherence levels <95%. In the absence of routine virological monitoring, interventions to prioritize timing of second-line ART based on CD4 counts and to support medication adherence will improve the treatment outcomes of patients on second-line ART in Vietnam.
