# Second-Line HIV Therapy Outcomes and Determinants of Mortality at the Largest HIV Referral Center in Southern Vietnam: Erratum

**DOI:** 10.1097/01.md.0000479540.74965.02

**Published:** 2016-01-08

In the article “Second-Line HIV Therapy Outcomes and Determinants of Mortality at the Largest HIV Referral Center in Southern Vietnam”,^1^ which appeared in Volume 94, Issue 43 of *Medicine*, Dr. Thao's degree was incorrectly listed as BSc. It should have read PhD. The article has since been corrected online.
